# Lectotypification of *Argyreia laotica* Gagnep. and *A. mekongensis* Gagnep. & Courchet (Convolvulaceae)

**DOI:** 10.3897/phytokeys.280.206333

**Published:** 2026-10-12

**Authors:** Tran Thai Vinh, Vu Kim Cong, Dang Thi Tham, Quach Van Hoi, Nong Van Duy

**Affiliations:** 1 Institute of Life Sciences, Vietnam Academy of Science and Technology, 116 Xo Viet Nghe Tinh, Lang Biang – Da Lat Ward, Lam Dong, Vietnam Institute of Life Sciences, Vietnam Academy of Science and Technology Lang Biang – Da Lat Ward Vietnam

**Keywords:** *
Argyreia
*, Convolvulaceae, Indochina, nomenclature, typification

## Abstract

The names *Argyreia laotica* Gagnep. and *A. mekongensis* Gagnep. & Courchet were published in 1915, with the original material consisting of syntypes. Examination of the available original material confirmed seven extant syntype specimens for *A. laotica* and seven for *A. mekongensis*. Lectotypes are designated for both names based on examination of their original material.

## Introduction

*Argyreia* Lour. is a genus of Convolvulaceae established by Loureiro (1790), based on *A. obtusifolia* Lour. It is characterized primarily by indehiscent fleshy or mealy berries and is distributed throughout continental Asia, including Southeast Asia, the Indian Subcontinent, and China (Staples and Traiperm 2017; Lawand and Shimpale 2024). At present, approximately 144 species are accepted worldwide (POWO 2026).

Recent taxonomic and nomenclatural studies have improved the circumscription and typification of numerous names in *Argyreia* (Staples and Traiperm 2017; Lawand and Shimpale 2024). Nevertheless, several names published from Indochina during the early twentieth century remain inadequately typified. During our nomenclatural study of *Argyreia* in Indochina, we examined the available collections and relevant protologues and found that two species, *Argyreia laotica* Gagnep. and *A. mekongensis* Gagnep. & Courchet, were described based on multiple specimens or gatherings, without the designation of a specific specimen as the nomenclatural type. Lectotypification is therefore needed to fix the application of these names. Here, we designate lectotypes for both species and document the associated original material.

## Materials and methods

The protologues of *Argyreia laotica* and *A. mekongensis* were consulted in *Notulae Systematicae* (Paris) 3 (Gagnepain 1915). Original material was examined from high-resolution digital images available through JSTOR Global Plants (https://plants.jstor.org/) and digitized collections of the Muséum National d’Histoire Naturelle, Paris (P) (https://science.mnhn.fr/), Royal Botanic Garden Edinburgh (E) (https://data.rbge.org.uk/), the Natural History Museum, London (BM) (https://data.nhm.ac.uk/), Royal Botanic Gardens, Kew (K) (https://data.kew.org/) and Conservatoire et Jardin botaniques de Genève (G) (https://www.ville-ge.ch/musinfo/bd/cjb/). Herbarium acronyms follow Thiers (2026 onwards).

Nomenclatural information was verified using the International Plant Names Index (IPNI; https://www.ipni.org/), Plants of the World Online (POWO; https://powo.science.kew.org/), and Tropicos (https://www.tropicos.org/). Lectotypification was carried out in accordance with Art. 9.3 of the International Code of Nomenclature for algae, fungi, and plants (Turland et al. 2025).

All available original material was compared with the information provided in the protologues. These comparisons establish the connection to the protologues; completeness or diagnostic value alone does not establish original-material status. Among eligible specimens, we assessed preservation and the availability of vegetative and reproductive characters to select a lectotype that supports the application of each name.

## Typifications

### Argyreia laotica

Taxon classificationPlantaeSolanalesConvolvulaceae

Gagnep. Notul. Syst. (Paris) 3: 134 (1915).

83C8D613-31A9-5B5F-B949-AA3E2D4484B0

Ipomoea laotica (Gagnep.) J.R.I.Wood & Scotland. Nat. Plants 5(11): 1136–1144 (suppl.: 28) (2019).

#### Type material.

Laos. Expédition du Mé-kong, Kemmarath, *C. Thorel 3286* (lectotype, designated here: P [P00392012]; isolectotype: P [P00392013]).

#### Remaining syntypes.

Laos. *M. Massie s.n*. (P [P00392015, P00392016, P00392017]); *C. Dupuy 117* (P [P00392014, P02274584]).

#### Nomenclatural notes.

*Argyreia laotica* Gagnep. was described by Gagnepain (1915) in *Notulae Systematicae* based on three gatherings, *Massie s.n*., *Dupuy 117*, *Thorel 3286*, all of which constituted syntypes. In the original description, Gagnepain cited *Thorel* 3286 from “Kemmarath” and “Vien-thian”, while *Massie s.n*. and *Dupuy 117* were listed without locality data.

Staples and Traiperm (2017) reported seven extant syntype specimens preserved at the Muséum National d’Histoire Naturelle, Paris (P), comprising *Thorel 3286 p.p*. (P00392012, P00392013), *Massie s.n*. (P00392015, P00392016, P00392017), and *Dupuy 117* (P00392014, P02274584). The collector names and collection numbers recorded on these sheets correspond to the three gatherings cited in the protologue, supporting their identification as original material.

The gathering *Thorel 3286* is represented by two specimens P00392012 and P00392013. The label of P00392012 records “Kemmarath”, whereas that of P00392013 records “Vien-thian”; both localities are cited in the protologue. This gathering is therefore of particular nomenclatural significance because it provides the clearest geographical connection with the original description. Specimen P00392012 preserves sufficient vegetative and reproductive material to support the identification and application of the name and is therefore selected as the lectotype (Fig. 1). Specimen P00392013, representing a duplicate of the same gathering, is treated as an isolectotype.

**Figure 1.**
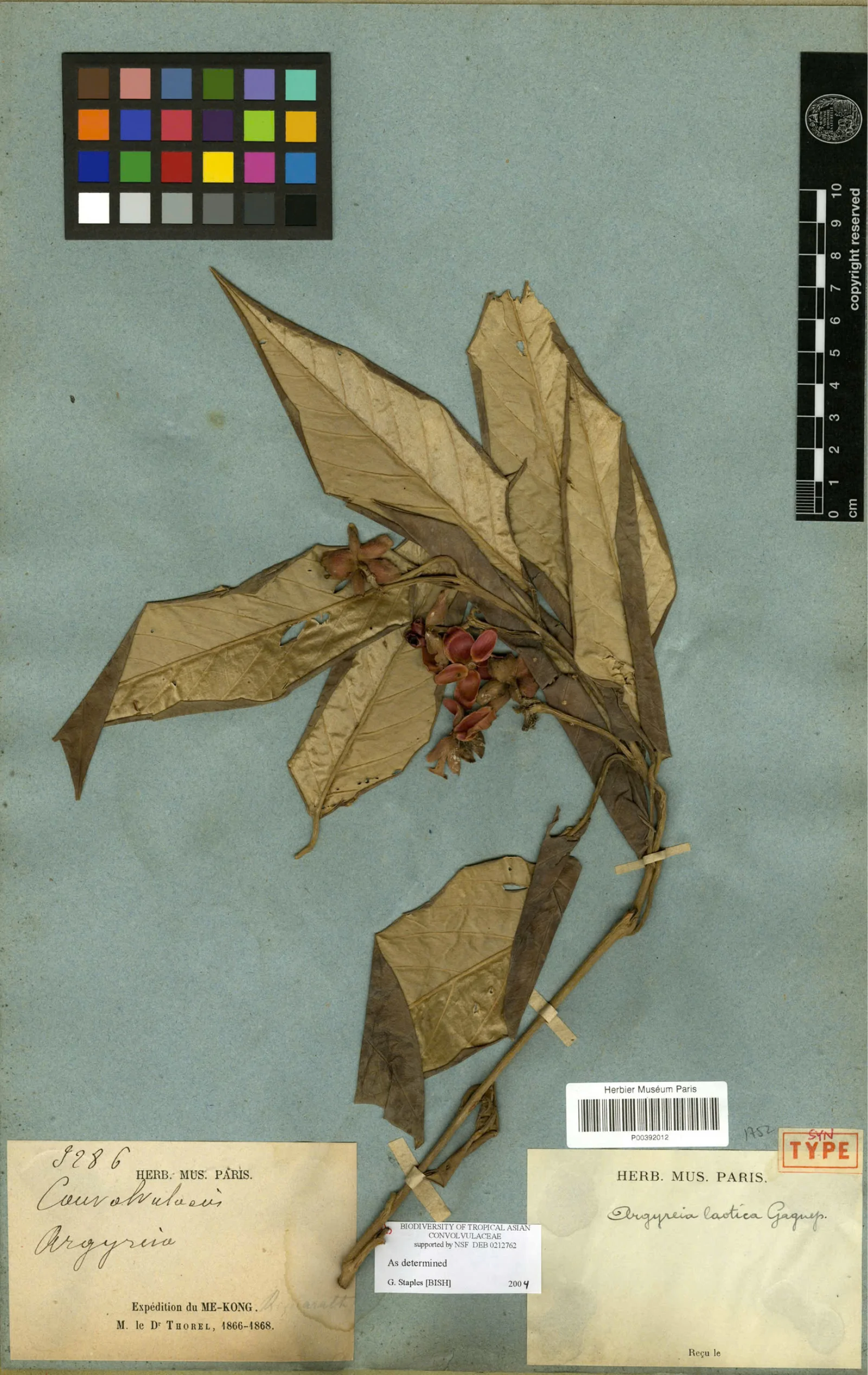
Lectotype of *Argyreia laotica* Gagnep., *Thorel 3286*, P00392012!. Photo: P.

### Argyreia mekongensis

Taxon classificationPlantaeSolanalesConvolvulaceae

Gagnep. & Courchet. Notul. Syst. (Paris) 3: 134 (1915)

59C02E9D-86DC-513E-9178-1449CB2194AA

Lettsomia mekongensis (Gagnep. & Courchet) Kerr. Fl. Siam 3(2): 34 (1954).
Ipomoea mekongensis (Gagnep. & Courchet) J.R.I.Wood & Scotland. Nat. Plants 5(11): 1136–1144 (suppl.: 28) (2019).

#### Type material.

Vietnam. Cochinchine, vers Baria, Bao-chiang, Gia-lau-me, *L. Pierre 2* (lectotype, designated here: E [E00273906]; isolectotypes: G [G00017128], K [K000830756]).

#### Remaining syntypes.

Vietnam. Cochinchine, vers Baria, Bao-chiang, Gia-lau-me, *L. Pierre 10* (K [K001081772]) • Cambodia. Stung-Treng, *C. Thorel 2150* (BM [BM000927943], E [E00273907], K [K000830755]).

#### Nomenclatural notes.

*Argyreia mekongensis* Gagnep. & Courchet was described by Gagnepain (1915) in *Notulae Systematicae* based on three gatherings, namely *Thorel 2150, Pierre 2*, and *Pierre 10*. In the protologue, the author cited *Thorel 2150* from “Stung-treng”, whereas *Pierre 2* and *Pierre* 10 were collected from “Cochinchine: vers Baria, Bao-chiang, Gia-lau-me”.

Staples and Traiperm (2017) documented several extant syntype specimens distributed among European herbaria, including *Thorel 2150* (BM000927943, E00273907, K000830755), *Pierre 2* (E00273906, G00017128, K000830756), and *Pierre 10* (K001081772). The original specimens at P cited in the protologue could not be located and were likewise reported as not found by Staples and Traiperm (2017). The collector names and collection numbers on the available specimens match the gatherings cited in the protologue; locality information is also consistent with the cited localities. These details support their identification as original material.

The gathering *Pierre 2* is represented by three specimens, G00017128, K000830756, and E00273906. This gathering is of particular nomenclatural significance because it originates from the locality cited in the protologue, and is represented by the most complete original material available. Among the examined specimens, E00273906 (Fig. 2) preserves abundant vegetative and reproductive material and exhibits the widest range of diagnostic characters among the available original material, including leaf morphology, indumentum, inflorescence structure, and floral features. It therefore provides the most suitable basis for the identification and application of the name and is selected as the lectotype. Specimens G00017128 and K000830756, representing duplicates of the same gathering, are treated as isolectotypes.

**Figure 2.**
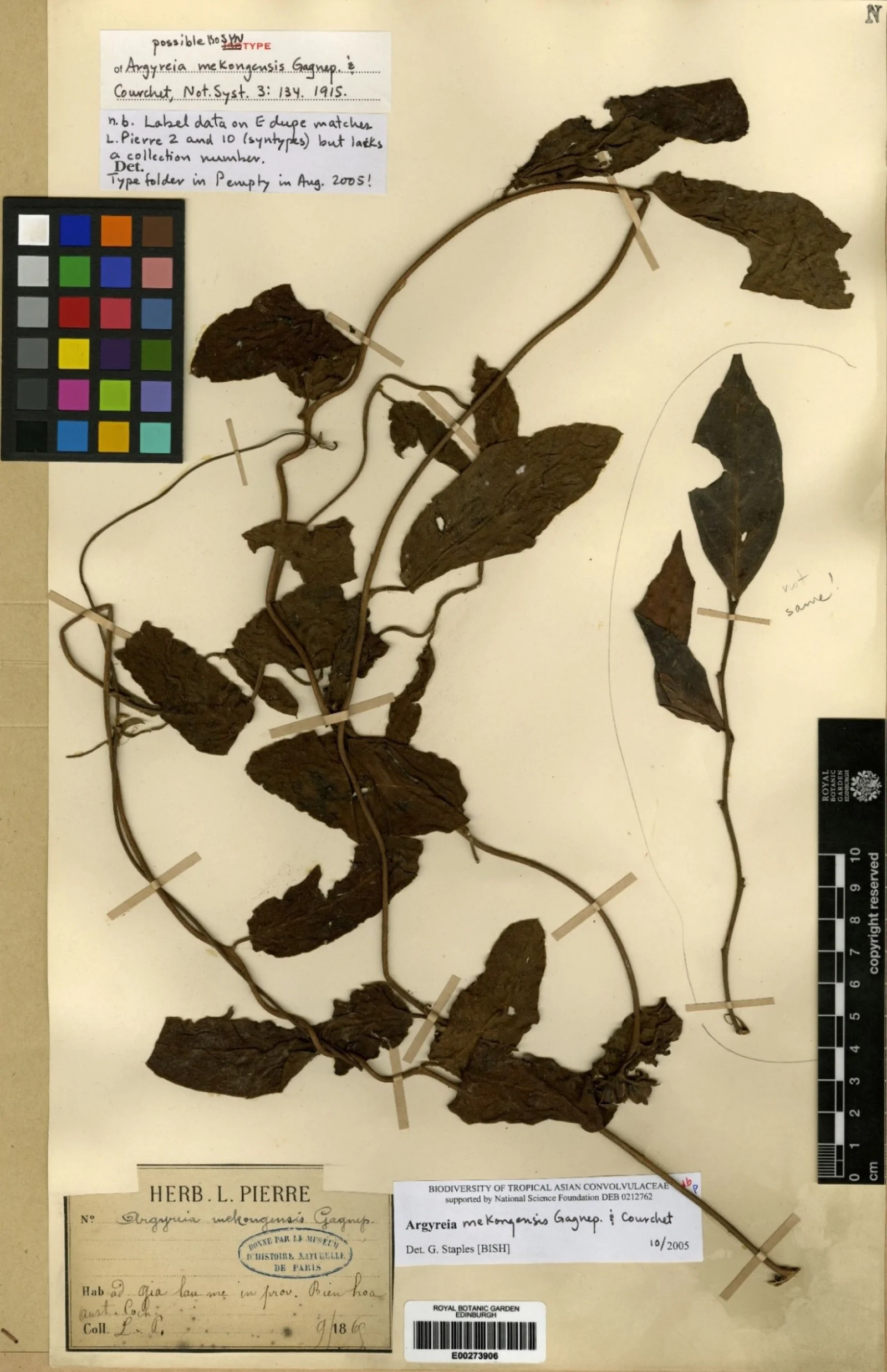
Lectotype of *Argyreia mekongensis* Gagnep. & Courchet, *Pierre 2*, E00273906!. Photo: E.

## Supplementary Material

XML Treatment for Argyreia laotica

XML Treatment for Argyreia mekongensis

## Additional information

### Artificial Intelligence (AI) use

The authors accept full responsibility for the content of the manuscript, including the disclosure of any use of AI.

No AI tools were used in the preparation of this manuscript.

### Author ORCIDs

T.T. Vinh https://orcid.org/0000-0001-6410-1423

V.K. Cong https://orcid.org/0009-0005-4979-0802

D.T. Tham https://orcid.org/0009-0008-8240-0126

Q.V. Hoi https://orcid.org/0000-0002-3691-941X

N.V. Duy https://orcid.org/0009-0009-9306-8357

### Data availability

All of the data that support the findings of this study are available in the main text.
